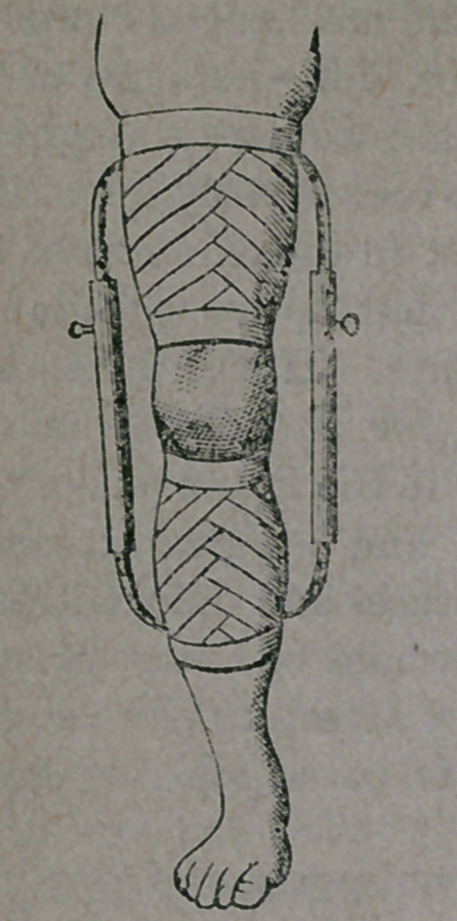# Our Joints

**Published:** 1874-01

**Authors:** 


### OUR JOINTS.

So little is known by the non-professional reader, of the mechanical contrivances in use by surgeons, for the cure of deformities of the body, that we have thought it not uninteresting to occasionally give illustrations of these means, in the columns of the Bistoury, in order to familiarize people with matters of this nature. If the public had better opportunities for learning what is constantly transpiring in the improvement of surgical instruments, and surgical treatment, of the various diseases and deformities of body and limb, they would be better prepared to adopt measures for arresting or removing many of the ailments to which we are continually subjected.

Perhaps no portion of our frame-work is called upon to perform more labor than our joints, and, when they cease or refuse to act, we at once discover that we are caused great inconvenience and pain.

When inflammation of a joint occurs, no matter from what cause, be it by reason of a blow, wound, rheumatism, or what not, it is highly important that the surfaces of the joint should not be allowed to come in contact, for in this manner the inflammation is increased and incouraged, often to the extent of the complete destruction of the joint.

How often we see young men and women hobbling about our streets, with one limb from three to six inches shorter than its fellow, with a correspondingly thick sole upon the shoe of the shortened limb, or an iron attachment to lengthen it out! These deformities are the result of "hip-joint-disease," in childhood, and could have been averted had parents known that means of cure were at hand. Commence the treatment of this form of disease *early*, as soon as the attending physician makes the discovery of its existance, and it will almost invariably yield to mechanical treatment. The orthopædic surgeon is supplied with means for "extending" or lifting the head of the bone from the socket, so relieving the pressure, while suitable means are employed to arrest the inflammation, preventing the formation of abscesses, and consequent destruction of the joint.

In the various diseases to which the knee-joint is subjected, similar appliances are used for preventing the surfaces of the joints from touching, affording the surgeon opportunity for curing the disease. How this can be accomplished will be understood from the illustration here given :

Suitable, non-yielding, and snugly fitting bands are placed about the limb, one above and another below the knee, and are better secured by means of a roller bandage. To these bands are attached loops, to which are secured the "extension" apparatus, consisting of a light, but strong steel frame, capable of throwing the joint apart to any desired extent. By this means, the inflamed surfaces of the joint are prevented from touching each other, preventing pressure, and so greatly assisting the surgeon in subduing the inflammation.

To such means as these, is the intelligent surgeon indebted for the astonishing cures that he is able to perform,—cures that could not possibly have been accomplished, save through their instrumentality.

## Figures and Tables

**Figure f1:**